# Long-term survival and prognostic factors for locally advanced renal cell carcinoma with renal vein tumor thrombus

**DOI:** 10.1186/s12885-019-5359-0

**Published:** 2019-02-13

**Authors:** Chuanzhen Cao, Xingang Bi, Jing Liang, Lin Li, Huijuan Zhang, Zhendong Xiao, Zejun Xiao, Jun Tian, Dong Wang, Kaopeng Guan, Changling Li, Jianhui Ma, Shan Zheng, Jianzhong Shou

**Affiliations:** 1 Department of Urology, National Cancer Center/National Clinical Research Center for Cancer/Cancer Hospital, Chinese Academy of Medical Sciences and Peking Union Medical College, Beijing, Panjiayuan Nanli 17#, Chaoyang District, 100021 People’s Republic of China; 2 Department of Urology, Civil Aviation General Hospital & Civil Aviation Clinical Medical College of Peking University, Beijing, 100123 China; 3 Department of Pathology, National Cancer Center/National Clinical Research Center for Cancer/Cancer Hospital, Chinese Academy of Medical Sciences and Peking Union Medical College, Beijing, Panjiayuan Nanli 17#, Chaoyang District, 100021 People’s Republic of China

**Keywords:** Carcinoma, Renal cell, Renal vein, Tumor thrombus, Prognosis

## Abstract

**Background:**

Previous related studies have mainly focused on renal cell carcinoma (RCC) with venous tumor thrombus, specifically inferior vena cava tumor thrombus with renal vein tumor thrombus (RVTT). However, only a few studies have focused on postoperative long-term survival of RCC patients exclusively with RVTT. Our aim was to investigate the independent prognostic factors for locally advanced RCC with RVTT in China.

**Methods:**

Patients with locally advanced RCC with RVTT were enrolled for the study from January 2000 to December 2015. All patients underwent radical nephrectomy. Survival analysis was estimated using Kaplan-Meier. Univariable and multivariable survival analyses were performed using COX. Patients were divided into high-risk, middle-risk, and low-risk groups based on independent prognostic factors and then analyzed for survival.

**Results:**

One hundred twenty-eight consecutive patients (103 men & 25 women) were enrolled with a median age of 61 years. Thrombi were all graded 0 using the Mayo system, of which 23 were friable. None of the thrombi detached during surgery. 121 patients were successfully followed up, with a median follow-up period of 47 months. Median overall survival was 127 months (95%CI: 101–153). The 5-year and 10-year cancer-specific survival (CSS) rate was 67.9 and 57.0%. 59 patients had recurrence with median time of 40 months. Friable thrombus, paraneoplastic syndrome (PNS), modified Fuhrman grade 3/4 and perirenal fat invasion were independent prognostic factors (*p* < 0.05). The 5-year CSS for the Low-risk group (no factors) was 100%, Middle-risk group (1–2 factors) was 68.6%, while the High-risk group (3–4 factors) was 0%.

**Conclusions:**

After radical surgery, RCC patients with RVTT had a relatively fair prognosis except for patients with friable thrombus, PNS, higher modified Fuhrman grade and perirenal fat invasion.

**Electronic supplementary material:**

The online version of this article (10.1186/s12885-019-5359-0) contains supplementary material, which is available to authorized users.

## Background

One biological characteristic of renal cell carcinoma (RCC) is venous system invasion. This is observed in 5–36% of RCC patients [1, 2]. There are two major types, (*i*) renal vein invasion and (*ii*) renal vein with inferior vena cava invasion. The former is the major subtype, accounting for 60–78% [3, 4]. Renal vein tumor thrombus (RVTT) is the primary manifestation for renal vein invasion.

The majority of previous studies have focused on RCC with venous tumor thrombus (VTT), which included inferior vena cava tumor thrombus (IVCTT) together with RVTT. Diagnosis is usually performed by post-surgical pathology [2, 3, 5–12]. The reported post-surgical survival data for RCC with VTT varies significantly. The 5-year overall survival rate ranges from 34.0 to 71.0% [8–12], while the survival information for RVTT patients could only be extracted from the above studies, which were 50.9 to 65.0% [10–12]. Several studies have indicated that RCC patients with vein tumor thrombus have poor survival, with lymph node invasion, distant metastasis, and invasion of the IVC wall being independent prognostic factors [2, 3, 5]. Based on a retrospective study of 174 RCC patients with IVCTT or RVTT, Bertini et al. [7] proposed that friable thrombus was an independent predictor for poor survival for both IVCTT and RVTT. However, IVCTT and RVTT are staged differently based on the American Joint Committee on Cancer (AJCC). Hence it is more reasonable to discuss RVTT and IVCTT separately. In addition, the majority of previous studies have focused on IVCTT in European and American patients (Caucasians), with only a few with Chinese ethnicity [13]. Of the studies that had patients with Chinese descent, friable RVTT was not included in the prognostic analysis. Future studies on the prognostic and risk factors for RCC with RVTT are needed. There are two major concerns regarding the studies performed on RCC with RVTT. First, only a few studies have focused on post-surgical long-term survival of RCC patients with RVTT. Second, no common consensus on adjuvant treatment after radical surgery has been proposed.

This is one of a series of studies that focused on Chinese RCC patients with RVTT. In this study, we focused on long-term survival and prognostic factors after radical surgery in Chinese patients with RCC (locally advanced) with RVTT. We constructed a risk model for long-term survival in these patients with the hope that it may help select the best treatment strategy for future patients.

## Methods

We enrolled RCC patients with RVTT from the National Cancer Center/Cancer Hospital, Chinese Academy of Medical Sciences (NCC/CHCAMS) from January 2000 to December 2015. This study was approved by the Ethics Committee of NCC/CHCAMS (ID Num: NCC2016YJC-08). Patient consent was not required. We evaluated long-term survival and developed a prognosis model for Chinese RCC patients with RVTT.

All patients enrolled in the study met the 5-key inclusion criteria: (*i*) histopathology-confirmation of RCC with RVTT (graded 0 tumor thrombus by Mayo classification), negative surgical margins and no evidence of residual disease; (*ii*) patients underwent both enhanced abdominal CT and kidney MRI (either at NCC/CHCAMS or another qualified hospital) for clinical staging that suggested clinical RVTT; (*iii*) chest CT and abdomen ultrasonography were performed to exclude metastasis before surgery. Bone imaging or brain MRI was performed if necessary; (*iv*) patients had no additional treatments before retroperitoneal radical nephrectomy plus embolectomy; (v) complete follow-up information.

Patient demographic information, medical history, symptoms and signs, imaging and laboratory results, and pathologic characteristics were obtained from the medical records. Paraneoplastic syndrome (PNS) was defined as a set of symptoms, that included laboratory abnormalities involving systemic effects from the tumor not related to distant spread, infection, nutritional deficiency or treatment, such as fever, anemia, hypercalcemia, hypertension, or emaciation. The four typical PNS were:moderate-severe anemia (Hb < 60 g/L), emaciation (loss of weight ≥ 5.0Kg in 3 months), hypercalcemia (Ca2+ > 2.75 mmol/L) and persistent fever [14].

Surgery was performed using standard methods for open or laparoscopic radical nephrectomy. Retroperitoneal lymphadenectomy was performed when regional lymph node enlargement was observed by imaging or during surgery. All histopathological slides were reviewed based on the 2016 WHO urinary system and male genital organs histological classification criterion [15]. For friable thrombus, three pathologists provided their individual reports. The Mayo classification was used to identify VTT stage [1]. Thrombus consistency was defined as solid when the thrombus appeared compact and cohesive on more than 90% of its surface, while friable thrombus were irregular with necrotic areas and fragmented [7]. Tumor staging for each patient was reviewed again based on the 2010 AJCC TNM staging criterion, and was blinded in the previous pathology report of each patient.

Prognosis was obtained through telephone follow-ups and Electronic Medical Record System. Clinical and radiologic assessments during follow-up were based on the NCCN guidelines [16]. In brief, physical examination, blood sampling and imaging examinations were performed every 3 months for two years after surgery, and then every 6 months or annually afterwards. Telephone follow-ups included information on treatments after surgery, time of recurrence or metastasis, cause and time of death. All follow-ups were concluded on April 30th, 2016. The primary endpoint of the study was cancer-specific survival and was calculated from the time of surgery to the date of death related to RCC, or the last follow-up period.

### Selection of prognostic variables

Clinical, pathological and prognostic parameters were derived from literature review [5–7, 17, 18], and included age, gender, Body Mass Index (BMI), hematuria, osphyalgia, PNS, tumor laterality, tumor size, tumor necrosis, modified Fuhrman grade, perirenal fat invasion, metastasis of the regional lymph nodes, sarcomatoid differentiation, friable thrombus, blood transfusion, and adjuvant therapy. Cutoff values were selected based on our institutional-specific laboratory guidelines, median values or through related literature review.

### Statistical analysis

Survival time was defined as the number of months between surgery and the date of death or the last follow-up date. Kaplan-Meier was used to analyze cancer-specific survival (CSS) rates at 5 and 10 years after surgery, as well as median survival time. Univariable and multivariable COX proportional hazard regression models were used to determine prognostic and independent factors. Factors significant in univariable analysis were evaluated using multivariable models [19]. To stratify patients with significant risk of postoperative death, independent factors for prognosis were used to create a simple unweighted risk-factor model. The log-rank test was used to estimate differences between the curves and risk groups [19]. Statistical analysis was performed using SPSS version 21.0. Differences were considered statistically significant if *p* values were < 0.05.

## Results

We enrolled 4426 consecutive RCC patients from NCC/CHCAMS between January 2000 to December 2015, with 128 patients meeting our inclusion criteria. Clinical-pathological characteristics of the selected patients are summarized in Table 1. The median age was 61 years and 80.5% were males (M:F = 103:25). The major clinical symptoms were hematuria (41.4%) and asymptomatic renal mass diagnosed through physical examination (42.9%). Typical PNS was observed in 18 patients (14.1%).

**Table 1:** Patient characteristics and descriptive statistics

Variable	All patients (*n* = 128)
Median Age, years (range)	61.0 (34.0–87.0)
Sex, n (%)	
Male	103 (80.5)
Female	25 (19.5)
Median Body Mass Index (BMI), Kg/m^2^ (range)	24.7 (17.1–35.9)
Laterality, n (%)	
Right	60 (46.9)
Left	68 (53.1)
Clinical Symptoms, n (%)	
Hematuria	53 (41.4)
Osphyalgia	17 (13.3)
Paraneoplastic Syndrome (Fever, Anemia, Hypercalcemia, Emaciation)	18 (14.1)
Asymptomatic	55 (42.9)
MaximumTumor Size, cm (range)	7.4 (2.7~19.0)
Perirenal Fat Invasion, n (%)	
Yes	52 (40.6)
No	76 (59.4)
Tumor Necrosis, n (%)	
Yes	33 (25.8)
No	95 (74.2)
pN+, n (%)	
Yes	9 (7.0)
No	121 (93.0)
Pathological Type, (%)	
Clear Cell Carcinoma	126 (98.4)
Chromophobe Cell Carcinoma	1 (0.8)
Type II Papillary Cell Carcinoma	1 (0.8)
Fuhrman Grade (Clear Cell Carcinoma), n (%)	
G1	7 (5.6)
G2	46 (36.5)
G3	52 (41.3)
G4	21 (16.6)
Sarcomatoid Differentiation, n (%)	
Yes	26 (20.8)
No	102 (79.2)
Friable RVTT, n (%)	
Yes	23 (18.0)
No	105 (82.0)
Median Bleeding Volume, ml (range)	150 (30~4000)
Median Volume of Blood Transfusion, ml (range)	1200 (200~4500)
Postoperative Adjuvant Therapy, n (%)	
Cytokine or Targeted Therapy	60 (46.9)
Active surveillance	68 (53.1)
Postoperative Local Recurrence/Metastasis, n (%)	
Lung Metastasis	39 (30.5)
Bone Metastasis	7 (5.5)
Local Recurrence	4 (3.1)
Thyroid Metastasis	2 (1.6)
Brain Metastasis	1 (0.8)
Scalp Metastasis	1 (0.8)
Abdominal Wall Metastasis	1 (0.8)
Lymph Node Metastasis	1 (0.8)
Multiple Organ Metastasis	3 (2.3)

89 (69.5%) patients were diagnosed with RVTT before surgery, while the remaining 39 (30.5%) patients were diagnosed with RVTT after the post-surgical pathology confirmation. Although no special pre-surgical treatments were performed for thrombus, none of them dislodged intraoperatively. During surgery, the distal end of the renal vein was clamped. This allowed for the complete removal of RVTT without tumor exposure, with negative incisal margins in the renal vein. This procedure differed from the intraoperative management for IVCTT.

The major pathological subtype was clear cell carcinoma (95.3%).

**Fig. 1:**
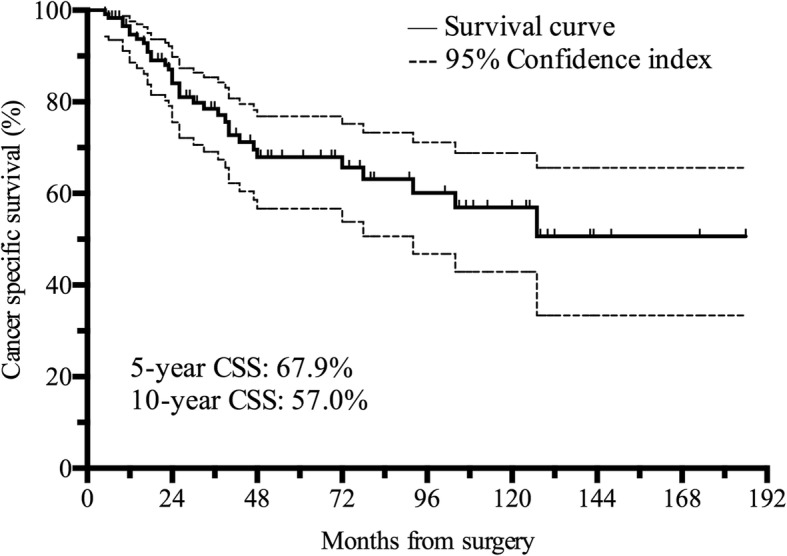
Kaplan-Meier plot of the 121 RVTT patients. Error bars were defined as 95% confidence index and denoted with a dash line. Median overall survival ± standard error (SE) [95% confidence interval (CI)] was 127 ± 13.4 (100.8–153.2) months. 5-year CSS was 67.9% and 10-year CSS was 57.0%

One hundred twenty-one patients were followed up successfully, with a median follow-up period of 47 months (7–186). Median survival time was 127 months (95%CI: 100.8~153.2). The 5-year cancer specific survival (5-year CSS) was 67.9% and 10-year CSS was 57.0% (Fig. 1). During the follow-up period, 59 (46.1%) patients had recurrence or distant metastasis with a median time of 40 months (2–108), of which, 20 (33.9%), 15 (25.4%), 9 (15.3%) and 15 (25.4%) received cytokine therapy, targeted therapy, surgery plus cytokine therapy and palliative therapy, respectively. 37 patients died, with 34 dying of tumor metastasis and 3 dying of cerebrovascular complications. Of the 84 patients alive during the follow-up period, 59 (70.2%) were cancer-free.

### Prognostic risk stratification model

We selected 15 factors for prognostic risk analysis (Table 2). Ten factors were candidate risk factors in univariable COX regression analysis (*p* < 0.05). We performed multivariable COX analysis for these ten factors. Modified Fuhrman grade 3/4 (*p* = 0.001, HR 5.194), PNS (*p* = 0.004, HR 3.613), friable RVTT (*p* = 0.006, HR 4.149) and perirenal fat invasion (*p* = 0.007, HR 3.032) were independent prognostic factors identified (Table 3).

**Table 2:** Univariable Cox analysis for the fifteen factors

Factors	No.	Median Survival Time (Months)	HR	95%CI	*p* value
Friable RVTT	23	24	16.065	6.531~39.518	< 0.001
Fuhrman Grade 3/4	69	47	8.278	3.357~20.412	< 0.001
Sarcomatoid Differentiation	26	24	5.435	2.764~10.689	< 0.001
Paraneoplastic Syndrome	16	24	5.031	2.398~10.554	< 0.001
Blood Transfusion	34	43	3.323	1.719~6.427	0.001
Perineal Fat Invasion	51	48	3.008	1.543~5.866	0.001
Tumor Size≥7 cm	69	49	3.491	1.587~7.681	0.002
Tumor Necrosis	30	43	2.246	1.121~4.501	0.022
pN+	9	26	2.779	1.072~7.204	0.035
BMI (< 24.7Kg/m^2^)	58	127	2.033	1.038~3.982	0.039
Tumor Side (Left)	63	92	0.556	0.282~1.099	0.092
Hematuria/Osphyalgia	69	78	1.736	0.853~3.533	0.128
Gender (Male)	96	186	1.446	0.676~3.092	0.341
Adjuvant Therapy	56	104	0.374	0.334~1.434	0.742
Age (> 60 yrs)	62	92	1.037	0.503~2.138	0.298

**Table 3:** Independent prognostic factors analyzed using multivariable COX model

Factors	β	SE	Wald	HR	95%CI	p
Fuhrman Grade 3/4	1.648	0.503	10.744	5.194	1.939~13.912	0.001
Paraneoplastic Syndrome	1.285	0.450	8.153	3.613	1.496~8.726	0.004
Friable RVTT	1.423	0.517	7.570	4.149	1.506~11.431	0.006
Perineal Fat Invasion	1.109	0.413	7.218	3.032	1.350~6.812	0.007

**Fig. 2:**
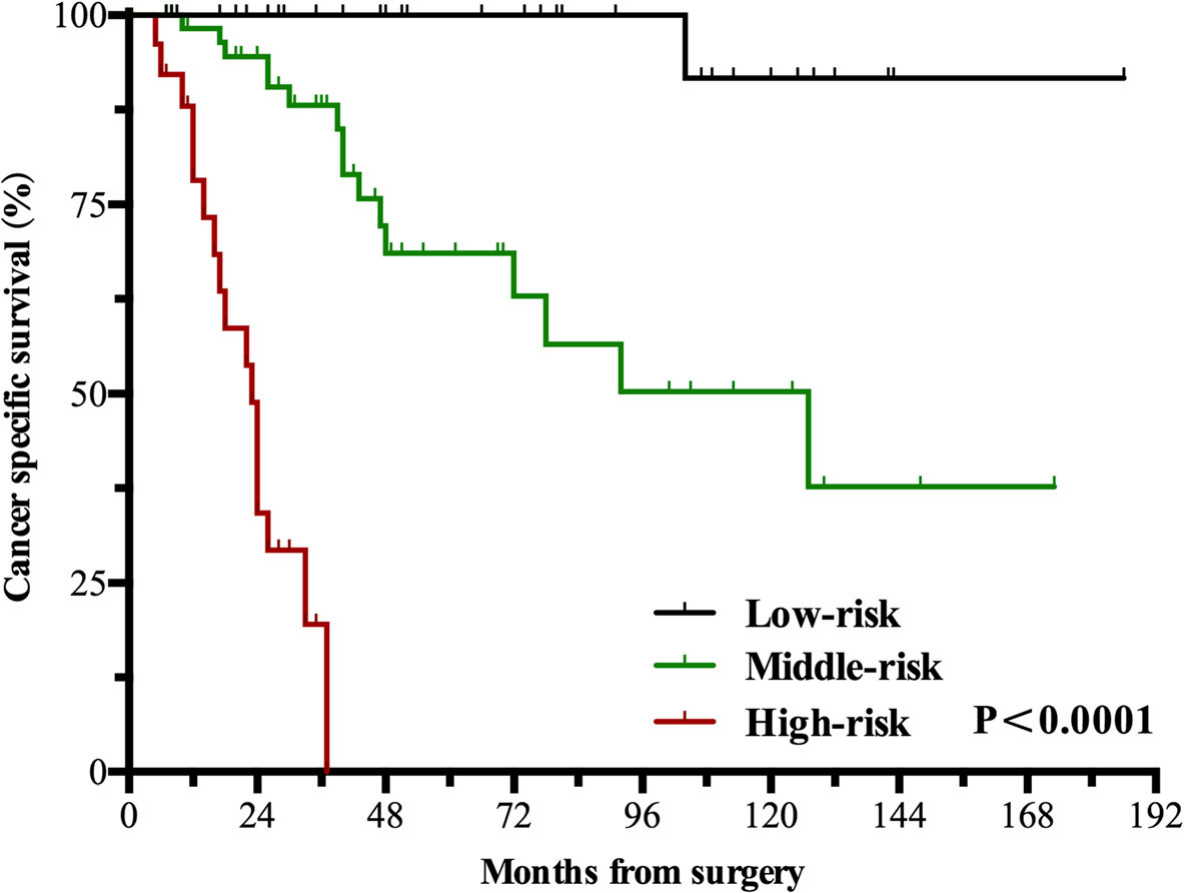
Kaplan-Meier plots with log-rank statistics for the three different risk groups. Patients in the High-risk group had significantly worse prognosis compared to the other two groups (*p* < 0.0001)

We constructed an unweighted prognostic risk stratification model for the 121 patients based on different combination numbers of the independent factors. The model stratified patients into three groups: low-risk (26 patients, 21.5%), middle-risk (60 patients, 49.6%) and high-risk (35 patients, 28.9%) group. The 5-year CSS of the low-risk (0 factor), middle-risk (1 or 2 factors) and high-risk group (3 or 4 factors) decreased sequentially by log-rank test (p<0.0001, Fig. 2).

## Discussion

In this study we aimed to determine the long-term survival and independent prognostic factors for patients with locally advanced RCC with RVTT in China. We found that RCC patients with RVTT treated with radical surgery had a relatively good prognosis, with 5-year CSS of 67.9%. Friable thrombus, PNS, higher modified Fuhrman grade and perirenal fat invasion were independent prognostic factors.

In our study, 5% of the 4426 consecutive RCC patients identified had VTT (128 RVTT vs 93 IVCTT), and was similar to studies performed previously in western countries [1, 2]. This indicated that Chinese and Caucasians had a similar propensity for VTT. However RVTT accounted for only 57.9%(128/221)of patients with VTT, which was lower compared to patients from western countries [3, 4].

Our criterion for patient enrollment was pathologic RVTT. In addition, we collected imaging data. We found that 39 patients with RVTT would have been missed if they were only diagnosed through imaging. This study is the first to compare pathologic RVTT with pre-surgical imaging information for RVTT. We hypothesized that tumors located in the middle pole or tumors having collateral vessels maybe one of the reasons. This suggests that urologists should pay special attention to the distal end of the renal vein during their diagnosis.

Most studies that have analyzed IVCTT with RVTT had reported different 5-year CSS. Hirono et al. [11] enrolled 292 RCC patients with 152 RVTT patients with or without metastasis and determined their 5-year CSS was 50.9%. Sidana et al. [12] retrospectively analyzed 132 RCC patients with 64 of them having RVTT. Their 5-year CSS was 65.0%. In our study, the 5-year CSS was 67.9%, and the 10-year CSS was 57.0% which has rarely been reported before. This indicates that locally advanced RCC patients with RVTT had relatively good prognosis (Fig. 1). This relatively good survival supports the AJCC TNM staging for RVTT. Hence, we think it is necessary to analyze RVTT separately in RCC patients.

Among the four independent factors in our study, the prognostic significance of modified Fuhrman grade and perirenal fat invasion were consistent with Western studies for IVCTT [5, 20]. However, the significance of thrombus consistency is controversial. Rene et al. [6] enrolled 413 RCC patients with IVCTT in their study that included patients with 225 solid and 188 friable thrombus. There was no statistical difference between the 5-year CSS for patients with friable versus solid tumor thrombus (*p* = 0.8). In a retrospective study cohort of 174 patients, friable thrombus was an independent predictor for survival and was associated with a significantly poorer CSS (p<0.001) [7]. However, there have been no RVTT studies that have focused on friable thrombus, although it is considered as a prognostic factor for IVCTT. Our study confirmed that friable RVTT was an independent prognostic factor for RCC patients with RVTT (*p* = 0.006). This suggests that friable thrombus may be associated with a higher risk of tumor haematogenous spread, and in clinically non-metastatic patients could be a higher risk for systemic progression. If our findings are confirmed in prospective studies, the friable description should be introduced into routine pathologic reports to provide additional information to guide adjuvant therapy.

It is estimated that the prevalence of PNS in patients diagnosed with RCC varies from 10 to 40% [14]. Moreira et al. [14] retrospectively analyzed 2865 RCC patients with PNS associated with poor CSS (*p* = 0.007), but PNS was not an independent factor. In our present study, patients were in the locally advanced stage, hence PNS was not sufficiently remarkable. We analyzed several typical symptoms (fever, emaciation) and laboratory abnormities (anemia, hypercalcemia). PNS was found to be an independent prognostic factor (*p* = 0.004). In addition, there were 52 patients with hypertension and 12 with abnormal liver function that were mainly caused by fatty liver diseases and could be resolved by hepatoprotective therapies pre-surgically. Hence these symptoms did not fit PNS clinically, and not considered in our analysis.

In a multiple-center study, Abel et al. [18] retrospectively analyzed 432 RCC with VTT, and divided them into low, middle and high risk groups on the basis of independent risk factors. The 5-year recurrence free survival rates of the three groups were statistically different (*p* < 0.001). Our study was a rare stratified analysis of VTT. We divided our patients into three risk-groups based on the independent prognostic factors for RCC with RVTT. The 5-year CSS of the high-risk group was significantly lower compared to the low-risk group (0% vs 100%). This indicated that the more prognostic factors patients had, the worse were their survival rates, and hence would need post-surgical treatment to improve their survivals. This is the first study to put forward risk stratification for RCC patients with RVTT. The prognostic factors were fewer compared to Abel’s. Hence our risk stratification had a better clinical operability. It provides patients who are classified as high-risk the opportunities to seek appropriate post-surgical adjuvant therapies.

At present, the standard of care for patients who had received nephrectomy for localized RCC is active surveillance [16]. In our study, of the 121 patients who were successfully followed up, 65 patients were on active surveillance, 38 patients accepted cytokine therapy including interleukin or interferon, and 18 patients accepted targeted therapy including Sorafenib or Sunitinib. The CSS of the three groups had no statistical difference (*p* = 0.673, χ^2^ = 0.793, Additional file 1: Figure S1). Hence accepting adjuvant therapy postoperatively had no significance in improving the survival of RCC patients with RVTT (Additional file 2: Figure S2). With phase III clinical trials of S-TRAC and PROTECT showing promising results [21–24], there has been great interest to investigate these agents in an adjuvant setting. Our study provides urologists with additional information to select high-risk patients for adjuvant therapies, while low risk patients should be on active surveillance.

There were several limitations to our study. We selected RCC patients with RVTT from NCC/CHCHAS to represent the whole Chinese population. Selection bias may exist during RCC patient enrollment in the selected hospital. However, the selection of this tertiary hospital was based on its ability for standardized diagnosis and treatment. Another limitation to the study was the inadequate follow-up period. The median recurrence time of 40 months suggested that the median follow-up period of 47 months was not sufficient. However, our follow-up period was longer compared to the 27 months follow-up period conducted in previous VTT analysis of Chinese patients [5]. We are continuing targeted patient follow-ups to supplement our data. In addition, the variables selected were based on the results from patients in Western countries and may not be ideal for the Chinese patient population. Our stratified model was only based on clinical data. Additional factors should be included to make our model more precise. Future multicenter studies to determine the benefit of our risk model should be performed. This will help investigate the appropriate post-surgical treatment strategies for patients classified as middle- and high-risk.

## Conclusions

After radical surgery, locally advanced RCC patients with RVTT had a relatively good prognosis except for those patients with friable thrombus, PNS, higher modified Fuhrman grade and perirenal fat invasion. Middle- and high-risk patients had the worse prognosis. We suggest that these patients be treated with adjuvant therapies to increase their chances of survival. As for low-risk patients, we propose to maintain active surveillance.

## Additional file

Additional file 1: **Figure S1**. CSS curves of different adjuvant therapies. Before putting cytokine and targeted therapy together as one factor (adjuvant therapy), we separately analyzed cytokine therapy and targeted therapy, the results indicated that they both had no significant benefits on CSS compared active surveillance (*p* = 0.673,χ^2^ = 0.793). (TIFF 130 kb)

Additional file 2: **Figure S2**. Univariable analysis for the factor of adjuvant therapy. In univariable cox analyses for the 15 factors, adjuvant therapy was not the significant factor of prognosis (*p* = 0.742). (TIFF 108 kb)

## Abbreviations

AJCC: American Joint Committee on Cancer
CAMS: Chinese Academy of Medical Sciences
CSS: Cancer-specific survival
IVCTT: Inferior vena cava tumor thrombus
NCC/CHCAMS: National Cancer Center/Cancer Hospital Chinese Academy of Medical Sciences
NCCN: National Comprehensive Cancer Network
PNS: Paraneoplastic syndrome
RCC: Renal cell carcinoma
RVTT: Renal vein tumor thrombus
VTT: Venous tumor thrombus
