# Supplementary figures and images for: Long-term survival and prognostic factors for locally advanced renal cell carcinoma with renal vein tumor thrombus
Source: BMC Cancer. 2019 Feb 13;19:144. doi: 10.1186/s12885-019-5359-0 (PMC6373083; doi:10.1186/s12885-019-5359-0)

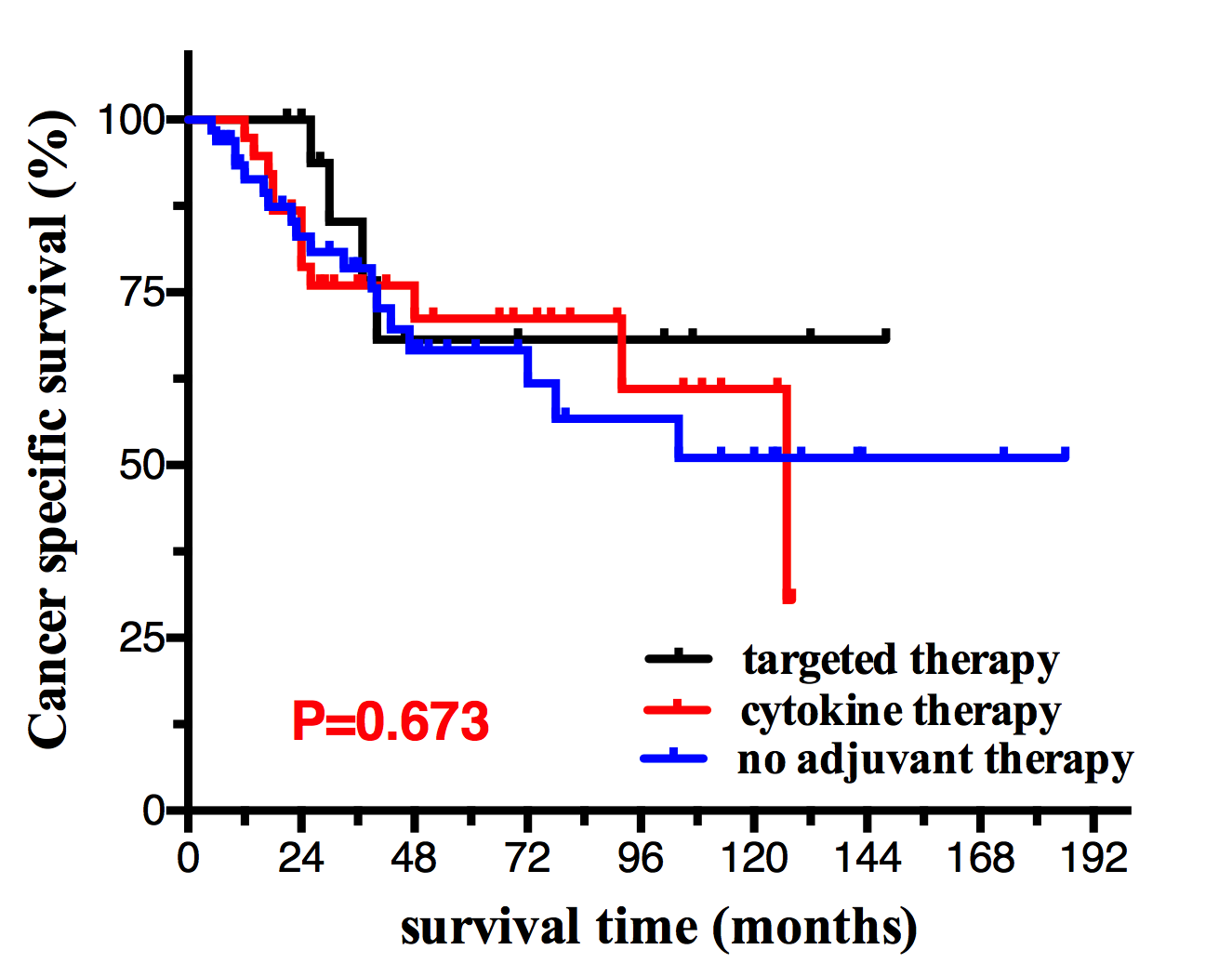

Supplement: Supplementary file 1 — Figure S1. CSS curves of different adjuvant therapies. Before putting cytokine and targeted therapy together as one factor (adjuvant therapy), we separately analyzed cytokine therapy and targeted therapy, the results indicated that they both had no significant benefits on CSS compared active surveillance (p = 0.673,χ2 = 0.793). (TIFF 130 kb) [file 12885_2019_5359_MOESM1_ESM.tiff]

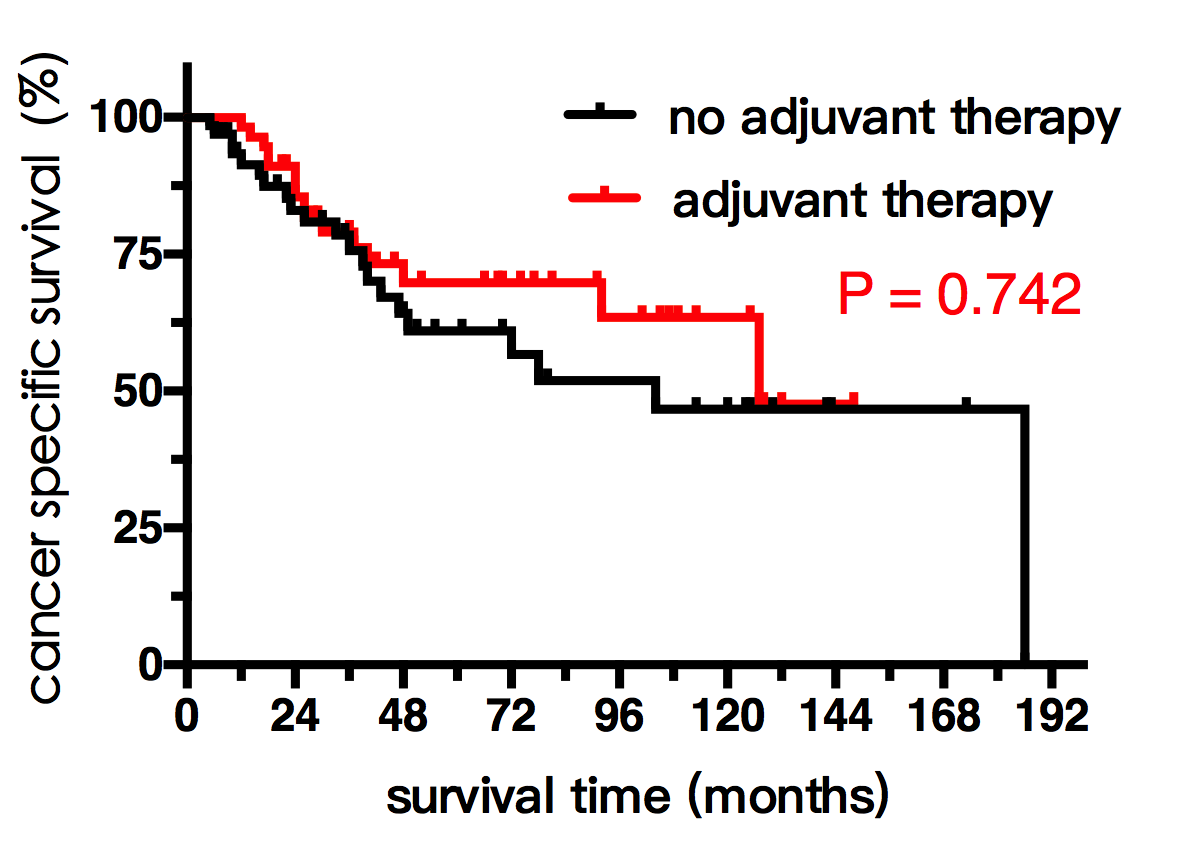

Supplement: Supplementary file 2 — Figure S2. Univariable analysis for the factor of adjuvant therapy. In univariable cox analyses for the 15 factors, adjuvant therapy was not the significant factor of prognosis (p = 0.742). (TIFF 108 kb) [file 12885_2019_5359_MOESM2_ESM.tiff]
